# Acute Compartment Syndrome and Intra-Abdominal Hypertension, Decompression, Current Pharmacotherapy, and Stable Gastric Pentadecapeptide BPC 157 Solution

**DOI:** 10.3390/ph18060866

**Published:** 2025-06-10

**Authors:** Predrag Sikiric, Sven Seiwerth, Anita Skrtic, Mario Staresinic, Sanja Strbe, Antonia Vuksic, Suncana Sikiric, Dinko Bekic, Toni Penovic, Dominik Drazenovic, Tomislav Becejac, Marijan Tepes, Zrinko Madzar, Luka Novosel, Lidija Beketic Oreskovic, Ivana Oreskovic, Mirjana Stupnisek, Alenka Boban Blagaic, Ivan Dobric

**Affiliations:** 1 Department of Pharmacology, School of Medicine, University of Zagreb, 10000 Zagreb, Croatiavuksic.antonia55@gmail.com (A.V.); drazenovic.dominik@gmail.com (D.D.); stupnisek@gmail.com (M.S.); 2 Department of Pathology, School of Medicine, University of Zagreb, 10000 Zagreb, Croatiaanita.skrtic@mef.hr (A.S.);; 3 Department of Surgery, School of Medicine, University of Zagreb, 10000 Zagreb, Croatia; 4 Department of Clinical Medicine, Faculty of Dental Medicine and Health Osijek, 31000 Osijek, Croatia; 5 Department of Diagnostic and Interventional Radiology, University Hospital Centre, 10000 Zagreb, Croatia

**Keywords:** abdominal compartment syndrome, intra-abdominal hypertension, severe multiorgan failure, pharmacotherapy, stable gastric pentadecapeptide BPC 157 therapy, critical conditions

## Abstract

In this study, pharmacotherapies of abdominal compartment syndrome (ACS) and intra-abdominal hypertension (IAH) in animal studies were reviewed from the perspective of ACS/IAH as failed cytoprotection issues, as non-specific injuries, and from the point of view of the cytoprotection concept as resolution. Therefore, this review challenges the unresolved theoretical and practical issues of severe multiorgan failure, acknowledged significance in clinics, and resolving outcomes (i.e., open abdomen). Generally, the reported agents not aligned with cytoprotection align with current pharmacotherapy limitations and have (non-)confirmed effectiveness, mostly in only one organ, mild/moderate IAH, prophylactic application, and provide only a tentative resolution. Contrarily, stable gastric pentadecapeptide BPC 157 therapy, as a novel and relevant cytoprotective mediator having pleiotropic beneficial effects, simultaneously resolves many targets, resolving established disturbances, specifically compression/ischemia (grade III and grade IV), and decompression/advanced reperfusion. BPC 157 therapy rapidly activates collateral bypassing pathways, and, in ACS and IAH, and later, in reperfusion, there is a “bypassing key” (i.e., azygos vein direct blood flow delivery). This serves to counteract multiorgan and vessel failure, including lesions and hemorrhages in the brain, heart, lung, liver, kidney and gastrointestinal tract, thrombosis, peripherally and centrally, intracranial (superior sagittal sinus), portal and caval hypertension and aortal hypotension, occlusion/occlusion-like syndrome, advanced Virchow triad circumstances, and free radical formation acting as a membrane stabilizer and free radical scavenger. Likewise, not only in ACS/IAH resolving, but also in other occlusion/occlusion-like syndromes, this “bypassing key” could be an effect of the essential endothelial cytoprotective capacity of BPC 157 and a particular modulatory effect on the NO-system, and a rescuing impact on vasomotor tone.

## 1. Introduction

Several recent reports indicate a particular role of cytoprotective stable gastric pentadecapeptide BPC 157 therapy in critical conditions. These were in therapy of severe vascular and multiorgan failure in occlusion/occlusion-like syndromes, induced with major vessel occlusion, peripheral [1,2,3,4,5] or central [6], several similar noxious procedures [7,8,9,10,11], and various damaging agents’ application [11,12,13,14,15,16,17]. Among them, specifically, there is the therapy of harms of abdominal compartment syndrome (ACS) and intra-abdominal hypertension (IAH), ischemia/compression [7], and decompression/advanced reperfusion [8]. From this viewpoint, this review highlights the unresolved theoretical and practical issues of ACS and IAH, acknowledged significance of severe multiorgan failure in clinics [18,19,20,21,22,23,24,25], and current resolving outcomes (i.e., open abdomen) [26,27,28,29]. Further focus was on animal studies, the limitations of pharmacotherapy attempts (mostly in rats) [30,31,32,33,34,35,36,37,38,39,40,41,42,43,44,45,46,47,48,49,50,51,52,53,54,55,56,57,58,59,60,61,62], and the therapeutic innovation advantage [7,8] of possible BPC 157 therapy applications.

In theory and practice, multiorgan failure during IAH and thereafter is a challenging problem [18,19,20,21,22,23,24,25,26,27,28,29], as the pleiotropic beneficial effects must simultaneously be achieved in opposing multiorgan failure during IAH. One possibility is simultaneously resolving multiorgan failure during and after IAH, achieved through the updated cytoprotection concept, targeting cell, epithelial, and endothelial protection [63,64,65,66]. However, as pointed out, it was not considered until recently for resolving the issue of complex multiorgan failure in ACS and IAH [7,8].

The cytoprotection concept was introduced initially in the rat stomach by Robert and Szabo, and since the early 1980s [67,68,69,70,71,72,73], has been extended into translation to other organ therapies via cytoprotective agent application (cytoprotection→organoprotection) [74,75,76]. For such translation, BPC 157, being native and capable of remaining stable in human gastric juice for more than 24 h, can be a cytoprotection mediator [63,64,65,66]. Thereby, there is a rapid pleiotropic beneficial effect [63,64,65,66], as mentioned, in overwhelming the severe multiorgan and vascular failure models (i.e., occlusion/occlusion-like syndrome [1,2,3,4,5,6,7,8,9,10,11,12,13,14,15,16,17], following occlusion of major vessels [1,2,3,4,5,6], peripherally [1,2,3,4,5] or centrally [6], noxious procedures [7,8,9,10,11] and noxious agents’ application [12,13,14,15,16,17]). There, this pleiotropic beneficial effect was updated consistently via activation of the collateral rescuing pathways (i.e., azygos vein direct blood flow delivery) to reestablish the reorganized blood flow. There were various noxious intra-abdominal procedures [63,64,65,66], i.e., inferior caval vein or superior mesenteric artery and/or vein occlusion [1,2,3,4], bile duct occlusion-induced acute pancreatitis [9], stomach perforation [10], all associated with ACS and IAH circumstances. In particular, considerable air insufflation leads to [7,8] grade III and grade IV IAH. Commonly, there was a counteraction of the multiple lesions and hemorrhage in the brain, heart, lung, liver, kidney, and gastrointestinal tract, and thrombosis, peripherally and centrally, intracranial (superior sagittal sinus), portal and caval hypertension, and aortal hypotension, via activation of collateral pathways (i.e., azygos vein direct blood flow delivery), advanced Virchow triad circumstances fully reversed [1,2,3,4,5,6,7,8,9,10,11,12,13,14,15,16,17].

Possibly, the long-standing lack of integration between cytoprotection and ACS/IAH management may stem from the limited efficacy of standard cytoprotective agents. Namely, they are effective primarily when used as pretreatment [67,68,69,70,71,72,73,74,75,76]. Another reason for the lack of integration may lie in the historical development of the cytoprotection concept, introduced in the early 1980s [67,68,69,70,71,72,73,74,75,76]. While IAH had been recognized earlier, in 1989, Fietsam introduced the term ACS [77,78]. Moreover, the full definition was even later created by guidelines (Abdominal Compartment Syndrome (WSACS), 2004). These guidelines define normal intra-abdominal pressure (IAP) in critically ill adults as approximately 5–7 mmHg. IAH is defined as a sustained or repeated IAP > 12 mmHg and is categorized into four grades: grade I (12–15 mmHg), grade II (16–20 mmHg), grade III (21–25 mmHg), and grade IV (>25 mmHg). ACS is defined as a sustained IAP > 20 mmHg associated with new organ dysfunction or failure [79].

## 2. Clinical Evidence

As pointed out [80], the generally acknowledged common goal is that treatment should aim to reduce risk and avoid permanent end-organ damage by reducing IAP and restoring regular systemic perfusion. Thereby, there is the improving of abdominal wall compliance (i.e., muscle relaxation, sedation, and neuromuscular blockade), evacuating of intra-luminal contents (i.e., nasogastric or rectal decompression, and prokinetic drugs), excessive abdominal fluid collections (i.e., paracentesis or percutaneous drainage), correcting positive fluid balance (diuretics, colloids or hypertonic fluids, hemodialysis and ultrafiltration) and supporting organs. Finally, there is abdominal decompression, the open abdomen therapy. Although proven to enhance renal function and urine output, and improve cardiac index and lung compliance [81], emergency laparotomy, whatever the type, is a high-risk operation; systemic inflammatory response syndrome, sepsis, and septic shock occur in 30% to 50% of EL patients, and mortality remains high [82,83,84,85,86,87,88,89,90,91].

To illustrate the complexity of the issue, decompressive laparotomy was commonly emphasized as a final solution. Additionally, the therapy problem arises as ACS occurs along with multifactorial disorders (i.e., typically attributed to critically unwell patients with trauma, burns, post-surgery, and massive ascites, but also gestation disorders, hernia, bulimia nervosa, COVID, and pancreatitis). There are also several predisposing factors (i.e., mechanical ventilation assistance, extracorporeal membrane oxygenation, elevated positive end-expiratory pressure, intestinal obstructions, excessive fluid replacement, major burns, and coagulopathies). Moreover, there is a high prevalence in intensive care unit patients (i.e., 59% medical and 41% surgical patients) [92,93]. Especially in grades III and IV, IAH occurs in over a third of patients and is associated with an increase in intra-abdominal sepsis, bleeding, renal failure, and death [94]. In addition, during laparoscopic surgery to provide intra-abdominal working space, permanent gas insufflation, commonly used, elevates IAP profoundly [95].

As indicated by [96], to illustrate clinical pathophysiology complex presentation, the most indicative may be the study of 102 severe traumatic brain injury patients with increased intra-abdominal, intrathoracic, and intracranial pressure, thereby, organ–organ interactions and resulting ‘multiple compartment syndrome’, and polycompartment syndromes [97]. Thus, as pointed out by Jacobs, with an increase in IAP, a negative impact on multiple organ systems occurs as a harmful network of the mutually interconnected disturbed systems, cardiovascular, respiratory, central nervous system, renal, and gastrointestinal tract [97,98,99,100,101,102,103,104,105,106,107,108]. Besides gastrointestinal failure (i.e., mesenteric vein compression, reduced perfusion, intestinal edema, bacterial translocation, and disturbances in the gut microbiome and immune function), it results in a general failure: There is reduced preload, increased afterload, lowered cardiac output, elevated diaphragm, decreased lung compliance, decreased lung functional residual capacity, intracranial hypertension, functional obstruction of cerebral venous outflow, and compression of both renal veins and arteries. The follow-up involves many specific syndromes, such as hepatic [109,110], renal [111,112,113], cardio-abdominal-renal [114,115], hepato-abdominal-renal [116,117], and hepato-abdominal-pulmonary [118,119] syndrome.

Therefore, the clinical evidence indicates that ACS is not a disease in itself [24,25]; Namely, it can have many causes and can develop within many disease processes, which can be multitude [24,26].

For basic studies, such a clinical multitude can represent an impending limitation (i.e., in basic studies, the elusive search for a single agent capable of addressing the complex and multifactorial nature of ACS (“magic bullet”) [120]) or an important insight for further therapy attempts [7,8]. In any case, to determine IAH and IAH consequences, such a multitude needs the largest possible extent of investigation, organs, and targets involved.

## 3. Basic Evidence

The recent introduction of the stable gastric pentadecapeptide BPC 157 as a possible therapy for acute ACS [7,8] follows extensive basic research of many agents, primarily in rats [30,31,32,33,34,35,36,37,38,39,40,41,42,43,44,45,46,47,48,49,50,51,52,53,54,55,56,57,58,59,60,61,62] (Table 1), but also in other species [121,122,123,124,125,126,127,128,129,130,131,132] (Table 2). Likewise, for BPC 157 therapy, this implicates a step forward and high effectiveness (i.e., effective in the 10 µg–10 ng/kg range) in terms of treatment. Furthermore, in toxicology studies, BPC 157 exhibited a negative limit test, 2 g/kg iv or ig without adverse effects in mice, with no lethal dose (LD1) identified [63,64,65,66]. Later, it was effectively used in ulcerative colitis trials (phase II) without adverse effects [63,64,65,66,133,134]. Also without adverse effects are subsequent therapy studies of knee pain [135] and interstitial cystitis [136]. Although these clinical studies are scarce in comparison with animal data (i.e., lacked a large sample size, ethnic variation, and sham control group), they reflect a wide range of therapeutic benefits consistent with findings from animal models [63,64,65,66].

Note, the pharmacotherapy, the extensive basic research and many agents’ effectiveness so far done in many basic studies in rats [30,31,32,33,34,35,36,37,38,39,40,41,42,43,44,45,46,47,48,49,50,51,52,53,54,55,56,57,58,59,60,61,62] (Table 1) and other species [121,122,123,124,125,126,127,128,129,130,131,132] (Table 2) should be the most important for resolving the clinical multitude of treatment options. However, general ACS/IAH animal reviews [137,138] more closely analyzed the different animal models and pathophysiological effects of IAH, while the effectiveness of these agents remained far less analyzed.

Thus, approaching agent administration, such a multitude would be essential. The potential of resolving such a multitude would include how the various organs were investigated, how various agents were suggested to counteract ACS (Table 1 and Table 2), and various models, IAP values, and periods of insufflation/desufflation, ischemia/reperfusion, all investigated with a common tool to approach and resolve the consequences of IAH.

Therefore, to perceive the limitation or advantage of a particular agent’s application and its noted effectiveness, several highlights were pointed out as follows. Illustratively, the effectiveness of IAH depends on the organ(s) investigated, but evaluating IAH with multiple organ systems provides a more accurate and comprehensive assessment of its overall impact and therapeutic significance (see Section 3.1). Likewise, there is an important point that claim of agents’ effectiveness depends on the IAH level(s) investigated. Therefore, evaluation of the effectiveness of an agent in relation to multiple IAH levels should be mandatory for more accurate and comprehensive assessment of its effectiveness (see Section 3.2). Furthermore, as agents’ effectiveness depends on the organ or organs investigated, more accurate and comprehensive assessment would include more organs (see Section 3.3). Finally, there is the issue that the effectiveness of an agent depends on the time of its application. Consequently, prophylactic, preconditioning application to prevent or attenuate injury development and the application as a therapy in reversing already advanced injury should be tested (see Section 3.4). Also, there is the issue of the animal model that could determine the efficacy of the agent (see Section 3.5). On the other hand, no uniformity between the models used could hamper the comparison between the noted effectiveness of the agents.

### 3.1. IAH’s Effectiveness Depending on the Organ(s) Investigated

As a rule, with the agents investigated in the mentioned studies [30,31,32,33,34,35,36,37,38,39,40,41,42,43,44,45,46,47,48,49,50,51,52,53,54,55,56,57,58,59,60,61,62], the IAH effect was not postulated simultaneously on all or many organs. Instead, the studies included a particular investigation of each of the implemented organs, thus, organ by organ in separate studies, as an indicative target (but regularly using not more than 20 mmHg IAH). Thereby, specifically investigated in rat studies were the brain [139,140,141], heart [142,143,144], lung [145,146,147,148,149,150], liver [146,151,152,153,154,155,156,157], kidney [146,158,159], and gastrointestinal tract [154,160,161,162,163,164,165,166,167,168], while only a few studies simultaneously investigated more than one organ [146,154,165].

In general, this approach presents several limitations. At the very least, it may overlook the complex inter-organ interactions and systemic consequences of IAH. Likewise, separate studies for different organs make it difficult to directly compare outcomes or to establish a unified model of IAH-induced pathophysiology.

These limitations underscore the need for integrated, multiorgan investigations that better reflect the systemic nature of IAH. This issue seems to be resolved with BPC 157 therapy [7,8] (see Section 4).

### 3.2. Agents’ Effectiveness Depending on the IAH’s Level(s) Investigated

Likewise, to perceive the effectiveness of various agents used in the mentioned studies [30,31,32,33,34,35,36,37,38,39,40,41,42,43,44,45,46,47,48,49,50,51,52,53,54,55,56,57,58,59,60,61,62], the IAH used was moderate or mild, within grades I and II, and in a large majority of studies, the range of the IAH was confined to one level. Specifically used as an indicative target was an IAH of 8 mmHg [62], 12 mmHg [30,32,34,43,46,49], 12.5 mmHg [36,37], 13 mmHg [56], 14 mmHg [39,57], 15 mmHg [33,38,42,44,45,123,124,128,129,130,131,132], and 20 mmHg [35,40,41,47,52,53,54,55,58,59,60,125]. There were only a few studies of grade III, 25 mmHg [48] or 30 mmHg [122,126].

Such a general approach confined to low or moderate IAH levels indicates several general limitations, as low or moderate IAH levels do not fully capture the pathological spectrum encountered in severe clinical cases [94], potentially limiting the translational relevance of the findings. Furthermore, while only a few studies simultaneously investigated more than one IAH level [50,61,126,127], specifically, 7, 10, and 14 mmHg [50], 2, 4, 6, 8, 10, and 12 mmHg [61], and 11–16 cmH_2_O [127] and one study also used a higher range, 15, 20, 30, or 40 cmH_2_O IAP [126], the use of a single IAH level in most studies hinders the ability to assess dose-dependent or pressure-dependent responses, which are crucial for determining the therapeutic thresholds and safety margins of tested agents. These exceptions underscore the need for more comprehensive, multi-level IAH models to better evaluate therapeutic efficacy and to simulate clinical conditions more accurately.

Again, this issue seems to be resolved with BPC 157 therapy [7,8] given the simultanoeus investigation of IAH, grade III and grade IV, and consequences counteraction (see Section 4).

### 3.3. Agents’ Effectiveness Depending on the Organ(s) Investigated

Furthermore, within the conditions described in the mentioned studies [30,31,32,33,34,35,36,37,38,39,40,41,42,43,44,45,46,47,48,49,50,51,52,53,54,55,56,57,58,59,60,61,62], the various agents’ efficacy was commonly based mostly (Table 1) or only on counteraction of the oxidative stress and inflammatory response [46,56], but the noted effect was on one single organ, specifically, the intestine [30,36,37,40,52,55], lung [38,42,49], kidney [39,47,50,53,57,62], liver [33,34,61], or brain [32,41].

The other studies postulated the effect on the specific organ combinations, i.e., pancreas and intestinal tissues [31], respiratory, liver, and renal dysfunction [35], liver, small intestine, and lungs [43], liver, kidney, lung, and intestine [44], and kidneys, testicles, and prostate [45].

Thus, none of the studies focused on one or a few organ lesion assessments simultaneously investigated the full range of organ lesions. This can be relevant as therapies designed to treat one physiological concern may have detrimental effects on other parts of the body. This single- or limited-organ focus represents a key limitation for the translational relevance of preclinical findings. Thus, again, there is a need for integrative, multiorgan assessment strategies in future research. Again, this issue seems to be resolved with BPC 157 therapy [7,8], given the large range of organs simultaneously investigated during and after IAHs at grade III and grade IV (see Section 4).

### 3.4. Agents’ Effectiveness Considering Application Time

There is also a significant limitation regarding the timing of agent administration in the referenced studies [30,31,32,33,34,35,36,37,38,39,40,41,42,43,44,45,46,47,48,49,50,51,52,53,54,55,56,57,58,59,60,61,62]. The efficacy observed in these studies [30,31,32,33,34,35,36,37,38,39,40,41,42,43,44,45,46,47,48,49,50,51,52,53,54,55,56,57,58,59,60,61,62]—already limited by the use of only grade I and II IAH and a narrow range of organ involvement—appears closely tied to the timing of agent application. Notably, in nearly all studies, agents were administered *before* the induction of IAH.

Some agents required long preconditioning (i.e., days) [30,31,50,54], some required shorter preconditioning, i.e., hours [38,47,52,57] or minutes [33,34,46,49], or were given immediately before [39,43,44,45,55,56,62]. Otherwise, the agents were given immediately before decompression [35,58,59,60], and in one study, later, at 1 h after decompression [53].

Therefore, since they were administered before IAH introduction and not after IAH introduction, it seems that during IAH all agents would act particularly prophylactically, rather than therapeutically, to reverse the consequences of the presented IAH. Thus, in general, these studies offer limited insight into the agents’ capacity to reverse or mitigate organ damage once IAH is already established. Likewise, given the agents’ application later during IAH [35,58,59,60] but before decompression, except for application at the time of the advanced reperfusion [53], the therapeutic effect on reperfusion could not be directly established. Similarly, these studies in which agents were applied before decompression do not clearly distinguish whether the observed benefits stem from protection during sustained IAH, mitigation of reperfusion injury, or both. Thus, it could be that novel BPC 157 studies [7,8] would resolve the absence of studies focusing on post-injury (therapeutic) administration during or after established IAH, and consequently, also the translational potential for treating active or ongoing IAH-related organ dysfunction that otherwise remains uncertain.

### 3.5. Agents’ Effectiveness Considering the Animal Model

Another important limitation concerns the diversity and complexity of experimental models used to evaluate agent efficacy across studies [30,31,32,33,34,35,36,37,38,39,40,41,42,43,44,45,46,47,48,49,50,51,52,53,54,55,56,57,58,59,60,61,62]. To demonstrate agent’s efficacy [30,31,32,33,34,35,36,37,38,39,40,41,42,43,44,45,46,47,48,49,50,51,52,53,54,55,56,57,58,59,60,61,62], many studies used complex, specially composed models of antecedent IAH. Illustratively, there were portal hypertensions of 1 h, an abdominal restraint device, hemorrhaging and blood reinfusion [36,37], hemorrhagic shock/resuscitation [40,41], compensated and decompensated chronic heart failure, myocardial infarction [50]), and where IAH, throughout its introduction [32] or development [31,36,51], appeared in a quick succession of events. Notably, complexity may complicate the attribution of effects solely to IAH. Moreover, frequently, the groups appear to provide their particular model, i.e., Chang et al. [36,37] vs. Liu et al. [40,41]. Additionally, simple abdominal insufflation applied in most of the studies used various durations [30,33,34,38,39,42,43,44,45,46,47,49,52,53,54,55,56,57,58,59,60,61,62].

Therefore, the agents’ effects could hardly be comparable since there is no uniformity between the used models. Thus, the efficacy of the agent appears as such, providing the given outcome. Consequently, this would impact BPC 157 studies [7,8] as well.

In summary, a large number of patients exhibit more severe IAH, grades III and IV [94]. Consequently, the agents in basic studies should complain about these requirements. Thus, considering the therapeutic potential of the used agents in IAH and ACS in rats [30,31,32,33,34,35,36,37,38,39,40,41,42,43,44,45,46,47,48,49,50,51,52,53,54,55,56,57,58,59,60,61,62] (Table 1), and other species [121,122,123,124,125,126,127,128,129,130,131,132] (Table 2), the mentioned studies demonstrate the limited effectiveness of the applied agents (mostly prophylactic potential within moderate IAH, grade I and grade II, mostly one organ target).

Thus, the efficacy of the given agents in the mentioned studies [30,31,32,33,34,35,36,37,38,39,40,41,42,43,44,45,46,47,48,49,50,51,52,53,54,55,56,57,58,59,60,61,62,121,122,123,124,125,126,127,128,129,130,131,132] needs confirmation with more severe IAH, and on the considerably higher organ targets. However, these studies [30,31,32,33,34,35,36,37,38,39,40,41,42,43,44,45,46,47,48,49,50,51,52,53,54,55,56,57,58,59,60,61,62,121,122,123,124,125,126,127,128,129,130,131,132] revealed the effectiveness of a considerable number of agents belonging to different drug classes and sharing various essential actions [30,31,32,33,34,35,36,37,38,39,40,41,42,43,44,45,46,47,48,49,50,51,52,53,54,55,56,57,58,59,60,61,62,121,122,123,124,125,126,127,128,129,130,131,132]. This may suggest a non-specific action for resolution. Notably, the approach of the issues of IAH and ACS comes from multiple mechanistic points. Likely, this should be the point of consideration for the cytoprotection concept. Additionally, abdominal compartment syndrome is not a disease but rather a condition that can arise from various causes and develop within different disease processes [24]. Therefore, considering BPC 157 studies [7,8], it may be best addressed using general concepts such as cytoprotection [7,8,63,64,65,66], which could provide targeted solutions through the application of cytoprotective agents.

## 4. BPC 157 Evidence

Thus, BPC 157 evidence stands alongside the cytoprotection concept [7,8,63,64,65,66] (cytoprotection → organoprotection). The multitude is implemented in a general concept (cell protection, epithelial, and endothelial [67,68,69,70,71,72,73,74,75,76], and updated via the activation of rescuing collateral pathways [7,8,63,64,65,66]). Thus, the reliable potential resolution of such a multitude of IAH and ACS [7,8] would overwhelm the mentioned studies and currently suggested agents [30,31,32,33,34,35,36,37,38,39,40,41,42,43,44,45,46,47,48,49,50,51,52,53,54,55,56,57,58,59,60,61,62,121,122,123,124,125,126,127,128,129,130,131,132] (Table 1 and Table 2), which have shown limited and mostly prophylactic effects under mild IAH conditions and in single-organ models.

The reliable potential resolution of such a multitude would request a stable model, easily introduced and maintained, deeper investigation of severe IAH, grade III and grade IV, and a full range of the implemented organs, as all three body cavities communicate with each other; the brain, heart, lung, liver, kidney, and gastrointestinal tract lesions must be simultaneously investigated along with severe vascular failure. Also, there is an assessment of the intracranial, caval, and portal hypertension, aortal hypotension, and thrombosis, peripherally and centrally, of Virchow triad circumstances. The agent should be applied in an advanced injury stage, specifically in ischemia and specifically in reperfusion conditions, to illustrate the therapy’s potential to reverse already advanced either ischemic (compression) or reperfusion (decompression) lesions.

To these points, in BPC 157 studies [7,8], the time of the agent’s application was consistently accommodated (after 10 min of compression [7]; after 3 min of reperfusion [8]). Further, as the reliable potential (i.e., vs. severe IAH, grade III and grade IV [7], or vs. severe reperfusion [8]), resolving such a multitude of IAH and ACS [7], and reperfusion [8], at least in rats, stable gastric pentadecapeptide BPC 157 was the therapy used to mitigate critical conditions. Both BPC 157 regimens (µg and ng) had a similar therapeutic effect in all of the investigated protocols of ACS and reperfusion.

First, this was therapy for established primary abdominal compartment syndrome (25 mmHg/60 min, 30 mmHg/30 min, 40 mmHg/30 min, 50 mmHg/15 min) [7] (see Section 4.1).

Then, in a separate study, BPC 157 was the therapy for advanced reperfusion following maintained intra-abdominal hypertension (grade III and IV) in rats [8] (see Section 4.2).

### 4.1. BPC 157 Primary Abdominal Compartment Syndrome

**Figure 1:**
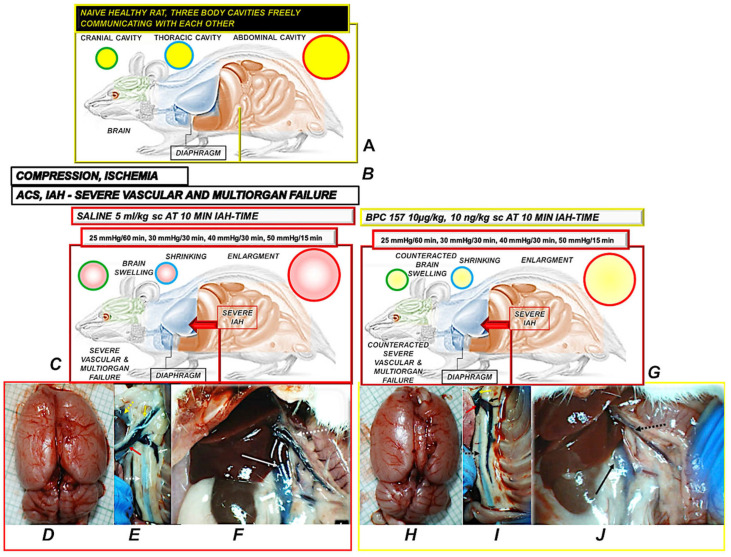
In normal rats (**A**) three body cavities (abdominal (red outlines), thoracic (blue outlines), and cranial (green outlines)) freely communicate (yellow circles indicating normal presentations). Along with maintained IAH and compression (**B**), rats had severe ACS and IAH, grade III and grade IV, ischemia/compression induced severe vascular and multiorgan failure, and severe occlusion/occlusion-like syndrome (**C**) (red circles indicating disturbed presentations, enlargement or shrinking), illustrated with brain swelling (**D**), collapsed azygos vein (**E**) and congested major vessels, inferior caval vein and superior mesenteric vein (**F**). With BPC 157 therapy at 10 min IAH-time, at already established ACS and IAH (**B**), counteracted was severe vascular and multiorgan failures (**G**), illustrated with counteracted brain swelling (**H**), activated azygos vein (**I**) (i.e., direct blood flow delivery via azygos vein), counteracted congestion of inferior caval vein and superior mesenteric vein, vein presentation close to normal (**J**) (arrows), counteraction of ischemia/compression-induced occlusion/occlusion-like syndrome as a whole by BPC 157 therapy (yellow shape outlines). This was coined with almost annihilated thrombosis and hemorrhage (i.e., intracerebral hemorrhage), peripherally and centrally, eliminated/attenuated venous hypertension (intracranial (superior sagittal sinus), portal, and caval) and aortal hypotension and counteraction of organ lesions (brain, heart, lung, liver, kidney and gastrointestinal tract) [7].

For compression studies, reliability is ascertained with the excessive IAH maintained during considerable periods, including 25 mmHg/60 min (or 25 mmHg/120 min), 30 mmHg/30 min, 40 mmHg/30 min, and 50 mmHg/15 min [7]. As such, in addition to gastrointestinal lesions and congestion, the ACS/IAH model ascertains the lesions in the brain (intracerebral/intraventricular hemorrhage), heart (congestion and infarctions), lung (hemorrhage), liver and kidney congestion, severe arrhythmias, intracranial (superior sagittal sinus)/caval/portal hypertension, aortal hypotension, widespread thrombosis, peripherally and centrally. Major vessels failed (congested (inferior caval vein, superior mesenteric vein), and collapsed (abdominal aorta, azygos vein)) (Figure 1) [7].

Thus, it is evident that in addition to the failed gastrointestinal tract, there were harms indued due to the mutually interconnected disturbed systems, i.e., cardiovascular, respiratory, central nervous system, renal, and gastrointestinal tract [7]. Consequently, all disturbances mentioned before, and specified by Jacobs [20] as reduced preload, increased afterload, lowered cardiac output, elevated diaphragm, decreased lung compliance, decreased lung functional residual capacity, intracranial hypertension, functional obstruction of cerebral venous outflow, and compression of both renal veins and arteries, occurred concurrently [7]. Note that these disturbances were estimated as advanced Virchow triad circumstances, a particular occlusion/occlusion-like syndrome (coagulation disturbances were implicated in ACS and IAH [169,170], and BPC 157 recovered thrombocyte function without interfering with the coagulation cascade [171,172]).

In addition, a pleiotropic beneficial effect of BPC 157 therapy was also noted in the similar multiorgan and vascular failure, and lesions presentation as an occlusion/occlusion-like syndrome was also described with peripheral [1,2,3,4,5] and central [6] major vessel occlusion, the application of severe noxious procedures [7,8,9,10,11], and noxious agents’ application [12,13,14,15,16,17]. In principle, such notation is consistent with IAH and ACS caused by various severe noxious events [94], as well as the effect of BPC 157 therapy in critical conditions [1,2,3,4,5,6,7,8,9,10,11,12,13,14,15,16,17], such as IAH and ACS. For the therapeutic effect, this could be a suitable cause-consequence lesion framework.

There is compelling evidence that BPC 157 therapy acts as a curative principle in rats with established permanent intra-abdominal hypertension [7]. The evidence for the reversal of blood vessel failure (as increased IAP compresses all blood vessels) was a full pleiotropic effect, first as a “bypassing key,” i.e., an activated azygos vein as a rescuing pathway, avoiding both the lung and liver [7]. A compelling illustration was also noted in the counteraction of Budd–Chiari syndrome (i.e., suprahepatic occlusion of the inferior caval vein) [173], combining the inferior caval vein and superior caval vein via direct blood delivery. Commonly, this means strengthening previously collapsed azygos vein (i.e., activated azygos vein direct blood flow delivery), previously congested inferior caval vein, and superior mesenteric vein as normal vein presentation, verified by assessing the corresponding increase or decrease in relative volume [1,2,3,4,5,6,7,8,9,10,11,12,13,14,15,16,17].

Thus, the evidence that the activated azygos vein reorganizes blood flow and instantly attenuates the consequences of maintained high intra-abdominal pressure, both peripherally and centrally, was general rat recovery by BPC 157 therapy, overwhelming the constantly maintained IAH, grade III and grade IV [7]. That included reduced/eliminated intracranial (superior sagittal sinus), portal, and caval hypertension, as well as aortal hypotension [7]. Additionally, severe ECG disturbances, such as severe bradycardia and ST-elevation, were also reversed. Microscopically, transmural hyperemia of the gastrointestinal tract, intestinal mucosa villi reduction, crypt reduction with focal denudation of superficial epithelia, and large bowel dilatation were all inhibited [7]. In the liver, BPC 157 reduced congestion and severe sinusoid enlargement. In the lung, a normal presentation was observed, with no alveolar membrane focal thickening and no lung congestion or edema, and severe intra-alveolar hemorrhage was absent. Moreover, severe heart congestion, subendocardial infarction, renal hemorrhage, brain edema, hemorrhage, and neural damage did not occur despite continuous severe IAH that is otherwise increasingly harmful and even deadly [7]. As additional proof appears, thrombosis reversal was evidenced peripherally and centrally, showing that advanced Virchow circumstances were fully reversed, peripherally and centrally [7]. With BPC 157 therapy, the brain (regularly, brain lesions, in the order cerebellum cortex > hypothalamus/thalamus > cerebral cortex while hippocampus, with increased lesion severity at higher intra-abdominal pressures, was particularly targeted) was preserved both grossly (absent brain swelling) and microscopically (consistent beneficial effect in all brain areas). The beneficial effect of BPC 157 acted against the full range of brain lesions, as the vicious course induced by high intra-abdominal pressure can be simultaneously initiated and perpetuated from different sites [7].

In addition, the mentioned BPC 157 follow-up through the cytoprotection concept maintaining epithelial and endothelial cell integrity as one of the cytoprotection mediators [63,64,65,66], BPC 157’s endothelial effects and its function as a “bypassing key” confronting with established IAH and ACS [7,8], were ascribed to its particular interaction with the nitric oxide (NO) system (for a review, see [174,175]). These BPC 157 actions maintain a plethora of interactions with the NO system. The evidence shows that this could be the undisturbed NO-system functioning. This is the capability to induce the release of NO of its own [176,177]. Furthermore, there is the counteraction of the adverse effects of NO-blockade [174,175] (i.e., L-NAME hypertension [176] and prothrombotic effect [178]). Likewise, there is the counteraction of the adverse effects of NO-overstimulation [174,175] (i.e., L-arginine hypotension [176] and anti-coagulant effect [178]). Finally, BPC 157 therapy specifically activates the Src-caveolin-1-endothelial NO synthase (eNOS) pathway [179,180,181], underscoring the essential impact of BPC 157 on vasomotor tone. BPC 157 acts as a membrane stabilizer and free radical scavenger, and counteracts leaky gut syndrome, as shown in gastrointestinal tract cytoprotective studies [182]. BPC 157 also has a curative effect due to interactions with several molecular pathways [180,181,182,183,184,185,186,187,188,189,190].

### 4.2. BPC 157 and Reperfusion After Decompression

**Figure 2:**
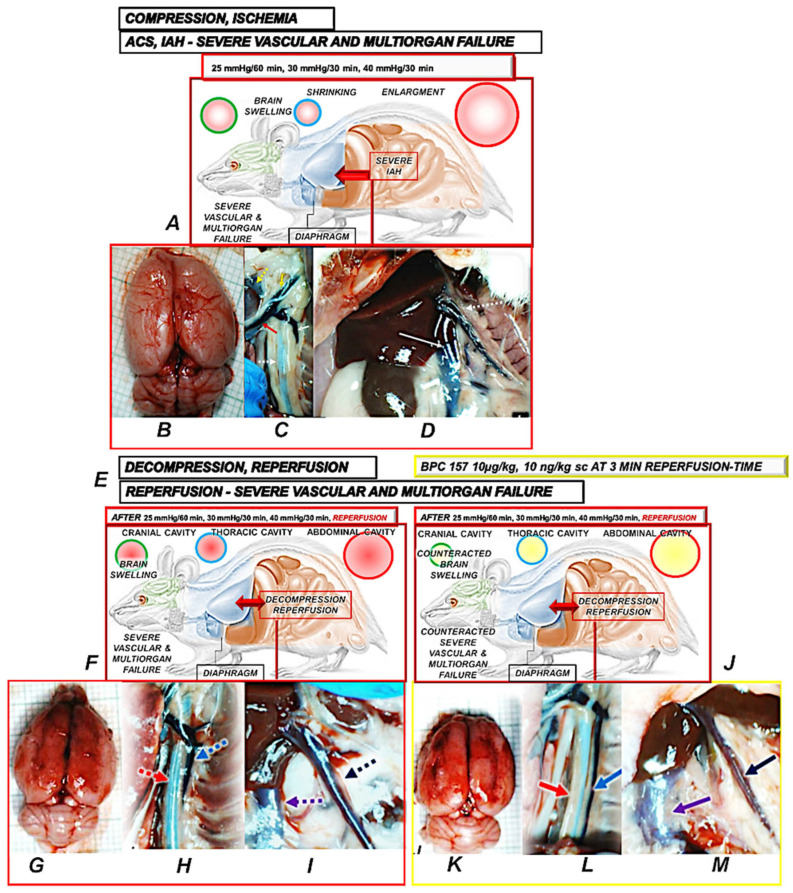
In rats that had severe ACS and IAH, grade III and grade IV, ischemia/compression induced severe vascular and multiorgan failure, and severe occlusion/occlusion-like syndrome (**A**), illustrated with brain swelling (**B**), collapsed azygos vein (**C**) and congested major vessels, inferior caval vein and superior mesenteric vein (**D**), after decompression, reperfusion (**E**) leads to further aggravation, severe vascular and multiorgan failure in controls (**F**), illustrated with severe brain swelling (**G**), collapsed azygos vein (**H**) and congested inferior caval vein and superior mesenteric vein (**I**) (arrows), decompression/reperfusion-induced occlusion/occlusion-like syndrome (red shape outlines). With BPC 157 therapy at 3 min reperfusion time, at already advanced reperfusion (**E**), counteracted was severe vascular and multiorgan failures (**J**), illustrated with counteracted brain swelling (**K**), activated azygos vein (**L**) (i.e., direct blood flow delivery via azygos vein), counteracted congestion of inferior caval vein and superior mesenteric vein, vein presentation close to normal (**M**) (arrows), counteraction of decompression/reperfusion-induced occlusion/occlusion-like syndrome as a whole by BPC 157 therapy (yellow shape outlines). This was coined with almost annihilated thrombosis and hemorrhage (i.e., intracerebral hemorrhage, eliminated/attenuated venous hypertension (intracranial (superior sagittal sinus), portal, and caval) and aortal hypotension and counteraction of organ lesions (brain, heart, lung, liver, kidney and gastrointestinal tract) and malondialdehyde (MDA) values (blood ˃ heart, lungs, liver, kidney ˃ brain, gastrointestinal tract) [8].

During the IAH and ACS, the failed abdominal, thoracic, and cranial cavities interact with each other [191]. Increased intra-abdominal pressure causes an increase in intracranial pressure [97,100,192,193]. Likewise, increased intra-abdominal pressure also increases intrathoracic pressure, which is rapidly transmitted up through the venous system, thereby further increasing intracranial pressure [97,100,192,193]. Thus, there is pleiotropic lesions presentation in abdominal, thoracic, and cranial cavities, and vice versa, and pleitropic beneficial effects in abdominal, thoracic, and cranial cavities [7,8]. Consequently, as a follow-up to its original beneficial effect, after the end of permanent intra-abdominal hypertension and decompression, during advanced reperfusion, BPC 157 therapy again has an immediate therapeutic effect and beneficial pleiotropic outcome, starting in the reperfusion conditions of already advanced severe lesions that would regularly finish toward the more perilous end (Figure 2) [8]. The noted pleiotropic beneficial findings along with reperfusion are compelling evidence of an even more rapid improvement of venous system function. This appears as an essential common point to prevent and reverse the noxious chain of events and attenuate all harmful consequences at any stage of the noxious course [7,8].

Therefore, BPC 157 therapy potential is applicable in ischemia [7] as well as in reperfusion [8]. Before decompression (calvariectomy, laparotomy), rats had long-lasting severe intra-abdominal hypertension, grade III (25 mmHg/60 min) (i) and grade IV (30 mmHg/30 min; 40 mmHg/30 min) (ii/iii), and severe occlusion/occlusion-like syndrome. Further worsening was caused by reperfusion for 60 min (i) or 30 min (ii/iii). Thus, the consistent applicability to both antecedent ischemia and advanced reperfusion stands from the same periods of ischemia and reperfusion used in quick succession [8].

For the therapeutic effect, a suitable cause-and-consequence lesion course was established, which was counteracted as a whole.

Otherwise, without therapy, aggravation occurred with severe vascular and multiorgan failure (brain, heart, liver, kidney, and gastrointestinal lesions), widespread thrombosis (peripherally and centrally), severe arrhythmias, intracranial (superior sagittal sinus) hypertension, portal and caval hypertension, and aortal hypotension were aggravated, and increased malondialdehyde (MDA) values regularly presented (blood ˃ heart, lungs, liver, kidney ˃ brain, gastrointestinal tract) [8].

Contrarily, with BPC 157 therapy, a similar effect as in compression studies [7], vascular recovery promptly occurred (i.e., congested inferior caval and superior mesenteric veins reversed to the normal vessel presentation, the collapsed azygos vein reversed to a fully functioning state, the inferior caval vein–superior caval vein shunt was recovered, and direct blood delivery returned, severe arrhythmias counteracted). This was coined with eliminated/attenuated venous hypertension (intracranial (superior sagittal sinus), portal, and caval) and aortal hypotension and counteraction of organ lesions and malondialdehyde (MDA) values (blood ˃ heart, lungs, liver, kidney ˃ brain, gastrointestinal tract) [8]. BPC 157 therapy almost annihilated thrombosis and hemorrhage (i.e., intracerebral hemorrhage). Thus, as proof, there was the counteracted general stasis, Virchow triad circumstances and reorganized blood flow rapidly counteracting the reperfusion course, and also reversing previous ischemia-course lesions, thus, inducing complete recovery, and decompression/reperfusion-induced occlusion/occlusion-like syndrome counteracted as a whole by BPC 157 therapy [8].

Finally, BPC 157 therapy in rats is likely to be translated to patients. As emphasized [63,64,65,66], it is quite distinctive from standard peptide therapy and the need for a delivery vehicle, as it always acts alone and can be a suited therapy application, whatever the route of application, and also via the per-oral route [63,64,65,66]. Namely, BPC 157, a stable gastric pentadecapeptide native that is stable in human gastric juice, can be released into circulation as a cytoprotective mediator and sent to distant organs [63,64,65,66]. Indeed, by in situ hybridization and immunostaining, BPC 157 was found in humans, in both adult and fetal tissues, gastrointestinal mucosa, lung bronchial epithelium, the epidermal layer of the skin, and kidney glomeruli [194], and thereby, exhibited a regulatory role. On the other hand, given ACS-IAH lesions and abdominal, thoracic, and cranial cavities interactions during ischemia—compression-occlusion/occlusion-like syndrome and decompression-reperfusion-occlusion/occlusion-like syndrome, and vice versa, pleiotropic beneficial BPC 157 therapy effects in abdominal, thoracic, and cranial cavities [7,8], BPC 157 was found to have an important role in brain–gut axis functioning [195]. Noteworthy, in addition to brain lesions in occlusion/occlusion-like syndromes, ACS and IAH studies [1,2,3,4,5,6,7,8,9,10,11,12,13,14,15,16,17], BPC 157 has been shown to reduce various brain lesions, such as trauma-induced brain injury, compression-induced spinal cord injury, stroke, and severe encephalopathies (NSAID overdose, neurotoxin cuprizone-induced multiple sclerosis in a rat model, and magnesium overdose) (for review see [195]). Accordingly, it is suggested to act as a neurotransmitter (or neurotransmitter-like) as it counteracts dopamine, serotonin, glutamate, GABA, adrenalin/noradrenalin, acetylcholine, and NO-system disturbances, specifically related to their receptors, both blockade and over-activity, destruction, depletion, tolerance, sensitization, and channel disturbances counteraction [196]. Additionally, BPC 157 therapy has a particular effect on muscle healing (i.e., striated, smooth, and heart muscle), and failed function recovery, which could particularly contribute to acute abdominal compartment recovery and improve abdominal wall compliance [197,198]. This can be part of a network of interconnected evidence [191,192], previously envisaged in the implementation of the cytoprotection effects (i.e., a cytoprotection mediator holds a response specifically related to preventing or recovering damage as such [67]). Possibly, the similar beneficial effects in other species (i.e., birds [199] and insects [200,201,202]) may suggest that BPC 157 may also have an extended regulatory physiologic role in bodily functions.

### 4.3. Final Remarks for BPC 157 and Other Agents Used in ACS/IAH Studies

Note that, for ACS and IAH issues, multiorgan failure is generally unresolved. Therefore, the cytoprotection concept, which encompasses a wide range of lesions being investigated and a broad pleiotropic range of beneficial effects [67,68,69,70,71,72,73,74,75,76], may be useful, particularly in basic studies. However, previous basic studies have not considered the cytoprotection concept and have missed a general approach in rats [30,31,32,33,34,35,36,37,38,39,40,41,42,43,44,45,46,47,48,49,50,51,52,53,54,55,56,57,58,59,60,61,62] (Table 1) and in other species [121,122,123,124,125,126,127,128,129,130,131,132]. Thus, their particular beneficial evidence (i.e., organ(s) being studied, IAH level(s) used, targets appointed) [30,31,32,33,34,35,36,37,38,39,40,41,42,43,44,45,46,47,48,49,50,51,52,53,54,55,56,57,58,59,60,61,62,121,122,123,124,125,126,127,128,129,130,131,132] has been summarized to illustrate the achievements and limitations of the investigated agents (Table 1 and Table 2).

To postulate the achievements and limitations of the investigated agents [7,8,30,31,32,33,34,35,36,37,38,39,40,41,42,43,44,45,46,47,48,49,50,51,52,53,54,55,56,57,58,59,60,61,62,121,122,123,124,125,126,127,128,129,130,131,132], skipping the analysis of the multiple systems that could be involved, the review adopted a more practical approach. This involved focusing on key representative findings (i.e., organ lesions, IAH level, time of application) while acknowledging the complexity of ACS pathophysiology. This holds a necessity for more organ investigation, and more IAH level investigation. Such an expansion is essential for a more accurate and comprehensive verification of therapeutic efficacy, particularly in the context of resolving multiorgan failure, which is central to the clinical manifestation of abdominal compartment syndrome (ACS).

Taken together, unlike other agents [30,31,32,33,34,35,36,37,38,39,40,41,42,43,44,45,46,47,48,49,50,51,52,53,54,55,56,57,58,59,60,61,62,121,122,123,124,125,126,127,128,129,130,131,132] with BPC 157 therapy [7,8], it seems that there are strong arguments. There is a large range of organs investigated, and lesions resolved. Likewise, there is a large range of IAH levels studied, resolving the severe grade III and grade IV. Furthermore, there is a demonstration of the rapid activation of rescuing collateral pathways (i.e., azygos vein direct flow delivery) as a bypassing key. Resolving occlusion/occlusion-like syndrome as a whole, means also attenuated/eliminated intracranial, caval, portal hypertension and aortal hypotension, hemorrhage and thrombosis, peripherally and centrally, with Virchow triad circumstances being fully reversed. This goes in ischemic (compression) studies [7]. Likewise, this also occurs in decompression (reperfusion) studies [8]. In all cases, therapeutic application occurs after injury induction (after establishing IAH, or after reperfusion). Thus, even with an established advanced injury course, there is a therapeutic effect on ACS and IAH. Accordingly, even with an established advanced injury course, there is a therapeutic effect on reperfusion. Additionally, there was a counteraction of the increased malondialdehyde (MDA) values in all organs [8]. Together, these would be very close to resolving the multiorgan failure in ACS and IAH as a whole, as well as in ischemia and reperfusion conditions.

Note that, unlike BPC 157 [7,8,63,64,65,66,194,195,196,197,198,199], the standard cytoprotective agents [67,68,69,70,71,72,73,74,75,76], as well as the agents used in the ACS and IAH studies [30,31,32,33,34,35,36,37,38,39,40,41,42,43,44,45,46,47,48,49,50,51,52,53,54,55,56,57,58,59,60,61,62,121,122,123,124,125,126,127,128,129,130,131,132], were always given before injury induction, i.e., before IAH induction, and they have a lower number of organs being investigated (mostly only one organ), and with only moderate or mild IAH (grade I and grade II). Also, unlike BPC 157 evidence, providing special studies in ischemia/compression, and special studies in decompression/reperfusion, the ACS and IAH studies with other agents combined together resulted in a shorter period of maintained IAH and a longer period after decompression [30,31,32,33,34,35,36,37,38,39,40,41,42,43,44,45,46,47,48,49,50,51,52,53,54,55,56,57,58,59,60,61,62,121,122,123,124,125,126,127,128,129,130,131,132]. Thereby, with all these agents, the particular effect of the agent on ACS and IAH, and the particular effect of the agent on reperfusion after decompression, both specifically remained undemonstrated [30,31,32,33,34,35,36,37,38,39,40,41,42,43,44,45,46,47,48,49,50,51,52,53,54,55,56,57,58,59,60,61,62,121,122,123,124,125,126,127,128,129,130,131,132].

## 5. Conclusions

As a framework, there is the crisis that occurred with the high IAP-induced syndrome, in which IAH simultaneously affected all abdominal vessels and organs for a considerable period and restrained the ability to recruit alternative pathways, and noxious course even more after decompression and reperfusion, such that a situation was deadly if therapy could be not initiated [7,8]. On the other hand, there is still no consensus regarding the most appropriate animal model for ACS [138,203]. In general, animal-to-human translation is a complex issue (for review see i.e., [204,205,206,207]), even when the wide range of translational success rates is consistently evidenced [206]. For example, the percentage of overall correct predictions reported by Litchfield is 74% when both rats and dogs are considered [207]. These data were used to calculate specificity (72%), sensitivity (76%), positive predictive value (68%), and negative predictive value (79%) [207].

Finally, since this paper at times implies strong therapeutic potential for BPC 157 in humans, it should be noted that, while encouraging, this should be carefully framed within the context of the early-stage nature of clinical evidence, given that the current support is primarily preclinical, and that further human trials are essential.

Thus, with all these caveats, some both conceptual and practical points have to be conclusively emphasized.

**Figure 3:**
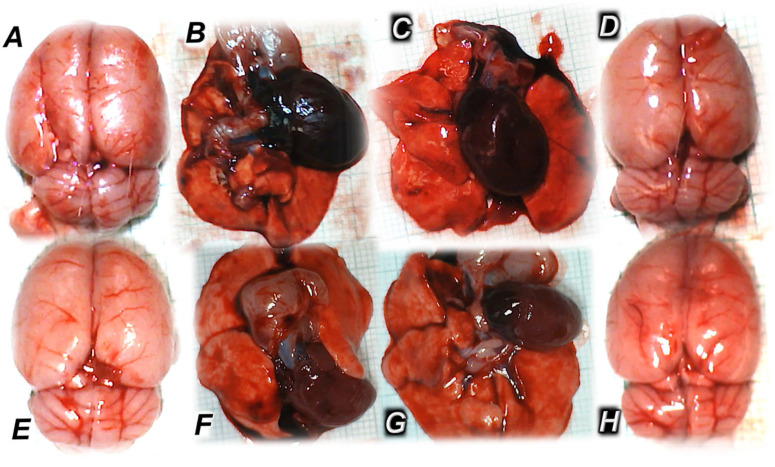
Illustrative presentation of the dynamics of the right-sided pneumothorax (**A**–**H**), (black italic capital letters for controls, (**A**,**B**,**E**,**F**)), and the BPC 157 therapeutic effect (white italic capital letters, (**C**,**D**,**G**,**H**), 10 µg/kg, 10 ng/kg ip immediately after pneumothorax induction (**C**,**D**) or 1 h after pneumothorax induction (**G**,**H**)) in rats. Immediately after pneumothorax induction (**A**–**D**). Illustratively, controls presented with brain swelling (**A**), collapsed lung (**B**), and counteraction occurred in BPC 157 rats, coutneraction of lung collapse (**C**) and counteraction of brain swelling (**D**). At 4 h after pneumothorax induction (**E**–**H**). As before, controls presented with brain swelling (**E**), collapsed lung (**F**), and counteraction occurred in BPC 157 rats, coutneraction of lung collapse (**G**) and counteraction of brain swelling (**H**). Pneumothorax induction at localization of fifth intercostal space, 1 cm from the spine, with coaxial biopsy needle inserted to depth of 1 cm into thorax, followed by transthoracic lung biopsy, and 3 cm^3^ of air instilled into the right heithorax, providing intrathoracic pressure of 30 mmHg (Koprivanac, personal communication, report in preparation).

Unlike the other agents’ studies implicated in ACS and IAH studies [30,31,32,33,34,35,36,37,38,39,40,41,42,43,44,45,46,47,48,49,50,51,52,53,54,55,56,57,58,59,60,61,62,121,122,123,124,125,126,127,128,129,130,131,132], for BPC 157 compression/ischemia and decompression/reperfusion, taking both as non-specific injurious events, and thereby, failed cytoprotection issues [67], the evidence shows BPC 157, as a novel and relevant cytoprotective mediator, has beneficial pleiotropic effects, rapidly activates collateral bypassing pathways, and alleviates vessel occlusion syndromes [7,8,63,64,65,66]. Therefore, the “bypassing key” (i.e., azygos vein direct blood flow delivery) serves to counteract multiorgan and vessel failure. The counteraction includes lesions and hemorrhages in the brain, heart, lung, liver, kidney, and gastrointestinal tract, thrombosis, peripherally and centrally, intracranial (superior sagittal sinus), portal and caval hypertension and aortal hypotension. Thus, counteraction includes occlusion/occlusion-like syndrome as a whole, advanced Virchow triad circumstances [7,8], and free radical formation acting as a membrane stabilizer and free radical scavenger [182,184]. Likewise, not only in acute abdominal compartment resolving [7,8], but also in other occlusion/occlusion-like syndromes [1,2,3,4,5,6,9,10,11,12,13,14,15,16,17], it has been theorized that this “bypassing key” appears to be an effect of the essential endothelial cytoprotective capacity of BPC 157 and a particular modulatory effect of the NO-system, and has a rescuing impact on vasomotor tone [174,175,176,177,178,179,180,181]. Possibly, this can be a prototype therapy for other compartment syndromes as well (i.e., pneumothorax) (Figure 3).

Thus, for ischemia-compression-occlusion/occlusion-like syndrome, conceptually, for ACS and IAH studies, this was the realization of the therapy effect simultaneously at the multiple locations affected by the severe compression (IAH occlusion/occlusion-like syndrome grade III and grade IV), given that the whole syndrome was counteracted as a whole [7]. Thereby, via BPC 157 therapy, an even more demanding therapy effect (vs. pleiotropic compression) than that in other occlusion/occlusion-like syndromes [1,2,3,4,5,6,9,10,11,12,13,14,15,16,17] —that BPC 157 therapy has consistently counteracted [1,2,3,4,5,6,9,10,11,12,13,14,15,16,17]—can be promptly achieved [7].

Thus, for decompression-reperfusion-occlusion/occlusion-like syndrome, even more complex circumstances are resolved. Severe ischemic conditions (compression) were instantly replaced by even more harmful reperfusion conditions (decompression) and post-decompression time (reperfusion, arising simultaneously from many sides after the end of compression, anticipating the imminent aggravation). There is also additional therapy potential for the activation of collateral pathways, i.e., azygos vein direct blood flow delivery, “bypassing key” that can be equally resolved [8].

Therefore, BPC 157 therapy in the post-decompression time resolved the worsening complexity of the advanced occlusion/occlusion-like syndrome [8]. As emphasized, antecedent to decompression, reperfusion, and therapy, there is a syndrome that barely survives by itself (i.e., severe bradycardia and ST elevation until asystole) during ischemia and compression, along with the consequent disturbances, thrombosis, stasis, vascular and multiorgan failure, and lesions, as mentioned before [7,8]. As such [8], it was also cured in reperfusion using the therapy, even in the worst conditions that occurred in reperfusion. Thus, BPC 157 therapy could be used in critical conditions, such as IAH and ACS, and subsequent reperfusion.

In conclusion, BPC 157 is proposed as a novel cytoprotective therapy that could effectively address the multifaceted challenges associated with ACS, IAH, reperfusion, and multiorgan failure, going beyond the limitations of current pharmacotherapy and surgical interventions like the open abdomen technique.

## Figures and Tables

**Table 1:** Agents’ effectiveness in ACS and IAH in rat studies presented in the timeline. Studies included based on Pubmed search using the basic term “abdominal compartment syndrome, rats”.

Ref.	Agent	IAH Procedure	Target	Outcome
[8]	Stable gastric pentadecapeptide BPC 157 (10 µg/kg, 10 ng/kg sc) given at 3 min reperfusion times	Reperfusion following maintained intra-abdominal hypertension (i) grade III: 25 mmHg/60 min or (ii), (iii) grade IV: (ii) 30 mmHg/30 min; (iii) 40 mmHg/30 min. Reperfusion for 60 min (i) or for 30 min (ii/iii)	Decompression/reperfusion-induced occlusion/occlusion-like syndrome: lesions and malondialdehyde (MDA) in the brain, heart, lung, liver, kidney, and gastrointestinal tract, thrombosis, hemorrhage, intracranial, portal, caval hypertension, aortal hypotension	Counteracted decompression/reperfusion-induced occlusion/occlusion-like syndrome as a whole. Counteracted: lesions and malondialdehyde (MDA) in the brain, heart, lung, liver, kidney, and gastrointestinal tract, thrombosis, hemorrhage, intracranial, portal, caval hypertension, aortal hypotension
[30]	L92, containing the single species *L. acidophilus*, 2.1 × 10^9^ CFU/kg/day, calculated as 200 × 10^8^ CFU for 7 days. Amino acid (AA) mixture treatment (trade name Elental) 25 g/kg/day for 7 days	90-min nitrogen pneumoperitoneum: IAP was 12 mm Hg	Colon histology, colonic reduced glutathione (GSH) and malondialdehyde (MDA) were used to evaluate the changes in oxidative responses. Colonic interleukin-1β (IL-1β) was measured to assess the inflammatory responses, 5-HT and 5-HIAA in plasma.	Orally gavaged *Lactobacillus acidophilus* L-92 (L92) and a mixture of AA in rats with induced IAH. The results showed that both L92 and AA pretreatments effectively mitigated IAH-induced intestinal damage. Interestingly, L92 but not AA prevented metagenomic changes induced by IAH.
[7]	Rats with intra-abdominal hypertension (grade III, grade IV) received BPC 157 (10 µg or 10 ng/kg sc) or saline (5 mL) after 10 min.	Intra-abdominal pressure in thiopental-anesthetized rats at 25 mmHg (60 min), 30 mmHg (30 min), 40 mmHg (30 min), 50 mmHg (15 min), and in esketamine-anesthetized rats at 25 mmHg for 120 min	Lesions in the brain, heart, lung, liver, kidney, and gastrointestinal tract, thrombosis, hemorrhage, intracranial, portal, caval hypertension, aortal hypotension	Counteracted occlusion/occlusion-like syndrome as a whole. Counteracted: lesions and malondialdehyde (MDA) in the brain, heart, lung, liver, kidney, and gastrointestinal tract, thrombosis, hemorrhage, intracranial, portal, caval hypertension, aortal hypotension
[31]	*C. butyricum* 1 × 109 colony-forming units (CFUs) of *C. butyricum*, Butyrate 100 mg/kg body weight of sodium butyrate in 1.0 mL of normal saline; for 10 days	Severe acute pancreatitis retrograde infusion, 4.5% sodium taurocholate (0.1 mL/100 g) 24 h after the operation, the IAP of each rat was determined	The plasma levels of several markers (amylase, diamine oxidase (DAO), fluorescein isothiocyanate (FITC)-dextran, tumor necrosis factor alpha (TNF-α), interleukin (IL)-6, IL-1β, IL-12, lipopolysaccharide (LPS)) and fecal butyric acid level were determined. The pancreas and intestine were examined using histology, and RT-PCR and Western blotting of intestinal tissues were used to measure the expression of six markers (tight junction proteins (zonula occludens protein-1 (ZO-1), claudin-1, claudin-2, occluding) matrix metalloproteinase 9 (MMP9), and TNF-α). The gut flora of the rats was examined by 16S rRNA sequencing	Rats that received oral *C. butyricum* or butyrate had reduced intestinal injury and plasma levels of DAO, LPS, and inflammatory cytokines.
[32]	Before blood was drawn, rats in the combined + bFGF group were treated with bFGF (10 μg/kg; intraperitoneal (IP) injection). FGFR1 antagonist, PD173074 (10 mg/kg; IP injection) 2 min before the administration of bFGF. PD98059 (ERK antagonist; 20 mg/kg; IP injection) 2 min before bFGF administration.	For moderate TBI, the impact depth was set as 2.0 mm, the dwell time was set as 100 ms, and the velocity was set as 3.5 m/s. Blood samples were taken (0.5 mL/min) within 10 min after the surgery, blood pressure was maintained at 30–40 mmHg for 1.5 h, then the reperfusion was induced by Ringer’s solution (30 mL/h, using an infusion pump). After 5 min, nitrogen was peritoneally injected until the IAP reached 12 mmHg.	Intracranial pressure (ICP) monitoring, brain water content, Evans blue permeability detection, immunofluorescence staining, real-time PCR, and Western blot analysis	The effects of bFGF on alleviating the rat BBB injuries were determined, indicating that bFGF regulated the expression levels of the tight junction (TJ), adhesion junction (AJ), matrix metalloproteinase (MMP), and IL-1β, as well as reduced BBB permeability, brain edema, and intracranial pressure. Moreover, the FGFR1 antagonist PD 173074 and the ERK antagonist PD 98059 decreased the protective effects of bFGF.
[33]	Hypodermic injection of hydrogen gas (0.2 mL/kg), and after 10 min they received an abdominal insufflation of CO_2_ for 90 min at an intra-abdominal pressure of 15 mmHg.	Abdominal insufflation of CO_2_ for 90 min at an intra-abdominal pressure of 15 mmHg.	Alanine aminotransferase (ALT) and aspartate aminotransferase (AST) were measured to evaluate liver function. Malondialdehyde (MDA), superoxide dismutase (SOD) and glutathione (GSH) content were measured to evaluate oxidative stress. Nuclear factor E2-related factor 2 (Nrf2) and Nrf2 downstream target genes, apoptosis-related genes and inflammatory cytokine mRNA and protein expression were detected. Liver injury was detected under the microscope.	Liver function, antioxidants content, inflammation and liver injury were improved after hydrogen preconditioning
[34]	i.v. glycine (1.5 mL, 300 mM) 10 min before pneumoperitoneum.	CO_2_ pneumoperitoneum (12 mmHg) for 90 min. Assessment at 1, 2, and 8 h after pneumoperitoneum	Transaminases, hepatic microcirculation, and phagocytosis of latex beads indexing both liver injury and KC activation were examined following pneumoperitoneum.	Glycine significantly decreased lactate dehydrogenase at 1 h and both aspartate aminotransferase and alanine aminotransferase at 2 h after pneumoperitoneum. In parallel, glycine significantly decreased both the rate of permanent adherence of leukocytes to the endothelium by up to 35% and the rate of phagocytosis by >50% compared to the control group.
[35]	CORM-3, CO donor, and GYY4137, H2S donor, were administered at the dose of 10 mg/kg and 50 mg/kg, respectively, via the carotid artery, just before decompression of the abdomen. Decompression 20–30 min.	2 h, an abdominal plaster cast and intraperitoneal CO_2_ insufflation at 20 mmHg. Decompression 20–30 min	Sinusoidal perfusion, inflammatory response and cell death were quantified in exteriorized livers. Respiratory, liver, and renal dysfunction was assessed biochemically.	Improved hepatic microvasculature, counteracted hepatic cell death, and inflammatory, metabolic, and renal dysfunction in a rat model of ACS
[36]	In the melatonin group (MT, n = 8), after blood reinfusion, rats were infused with melatonin (50 mg/kg), then resuscitated with Ringer solution (LR) (30 mL/h × 6 h). In the hypertonic saline group (HS, n = 8), after blood reinfusion, rats were infused with 7.5% hypertonic saline (HS) (6 ml/kg), then resuscitated with LR (30 mL/h × 6 h). In the hydroxyethyl starch group (HES, n = 8), after blood reinfusion, rats were infused with hydroxyethyl starch 130/0.4 (HES) (30 mL/kg), then resuscitated with LR (30 mL/h × 6 h).	Portal hypertension 1 h, abdominal restraint device, and hemorrhaging to mean arterial pressure (MAP) of 40 mmHg for 2 h. After blood reinfusion, the rats were treated with lactated Ringer solution (LR) (30 mL/h), for 6 h. The secondary IAH was determined by an elevation of 12.5 mmHg (170 mmH_2_O) of IVCP from the starting point.	The intestinal permeability, immunofluorescence of tight junction proteins, transmission electron microscopy, level of inflammatory mediators (TNF-a, IL-1β, IL-6) and of biochemical markers of oxidative stress (malondialdehyde, myeloperoxidase activity, and glutathione peroxidase) were assessed. Expressions of the protein kinase B (Akt) and of tight junction proteins were detected by Western blot.	Compared with LR, HS, and HES, melatonin was associated with less inflammatory and oxidative injury, less intestinal permeability and injury, and lower incidence of secondary IAH in this model. The salutary effect of melatonin in this model was associated with the upregulation of intestinal Akt phosphorylation.
[37]	Melatonin (50 mg/kg), and SB-203580 (10 μmol/kg) immediately after blood reinfusion	Portal hypertension 1 h, abdominal restraint device, and hemorrhaging to mean arterial pressure (MAP) of 40 mmHg for 2 h. After blood reinfusion, the rats were treated with lactated Ringer solution (LR) (30 mL/h), for 6 h. The secondary IAH was determined by an elevation of 12.5 mmHg (170 mmH_2_O) of IVCP from the starting point.	MAP, the inferior vena cava pressure and urine output were monitored. Intestine histopathological examination, immunofluorescence of tight junction proteins, and transmission electron microscopy were administered. Intestinal permeability, myeloperoxidase activity, malondialdehyde, glutathione peroxidase, and levels of TNF-a, IL-2, and IL-6. The expression of extracellular signal-regulated kinase, p38, c-Jun NH_2_-terminal kinase, translocation of nuclear factor kappa B subunit, signal transducers and activators of transcription and tight junction proteins were detected by Western blot.	Melatonin inhibited the inflammatory responses, decreased expression of p38 MAPK, attenuated intestinal injury, and prevented secondary IAH. Moreover, administration of SB203580 abolished the increase in p38 MAPK and also attenuated intestinal injury.
[38]	An arginase inhibitor 2(S)-amino-6-boronohexanoic acid (ABH) subcutaneous injection (5 mg/kg) 1 h before induction of pneumoperitoneum (insufflation to intraperitoneal pressure of 15 mmHg for 60 min)	Pneumoperitoneum (insufflation to intraperitoneal pressure of 15 mmHg for 60 min)	After desufflation, blood was collected to determine levels of plasma nitrite, NOS, inflammatory cytokines, and malondialdehyde, a marker of oxidative stress. Lung tissue was obtained for histological evaluation.	Pretreatment with an arginase inhibitor may protect against lung injury caused by pneumoperitoneum.
[39]	5 mg/kg of theophylline intraperitoneally before setting pneumoperitoneum model.	The pneumoperitoneum was generated by insufflating inside the abdomen by CO_2_ at 14 mmHg fixed pressure for 1 h, and desufflation was waited for 30 min	Urea, creatinine, cystatin-C, tissue and serum total antioxidant capacity, total oxidant capacity and oxidative stress index in two groups were measured and compared with each other. Apoptosis and histopathological conditions in the renal tissues were examined.	Lower cystatin-C levels in the group, where Theophylline was given, are suggestive of lower renal injury in this group. However, this opinion is interrogated as there is no difference in terms of tissue and serum TAS, TOS, OSI and urea values between the groups.
[40]	Selective melanocortin 4 receptor agonist RO27-3225 (180 μg/kg ip) 2 min before blood was drawn. The selective melanocortin 4 receptor antagonist HS024 (130 μg/kg) 2 min before the RO27 3225 administration. The nicotinic acetylcholine receptor antagonist chlorisondamine (250 μg/kg) 2 min before the RO27 3225 administration	IAH rat models were induced by hemorrhagic shock/resuscitation with the mean arterial pressure (MAP) maintained at 30 mm Hg for 90 min followed by the reinfusion of the withdrawn blood with lactated Ringer’s solution. Then, air was injected into the peritoneal cavity of the rats to maintain an intra-abdominal pressure of 20 mm Hg for 4 h.	Mean arterial pressure, reduced tumor necrosis factor-a, and interleukin-1b messenger RNA expression increased by IAH, histologic damage, and superoxide dismutase activity in the intestine, the levels of intestinal fatty acid-binding protein, intestinal edema and intestinal permeability, the expression of Rho-associated coiledecoil-containing protein kinase 1 and phosphorylated myosin light chain.	RO27-3225 restored mean arterial pressure, reduced tumor necrosis factor-a, and interleukin-1b messenger RNA expression increased by IAH, alleviated histologic damage, and improved superoxide dismutase activity in the intestine. Compared with the IAH group, the levels of intestinal fatty acid-binding protein, intestinal edema and intestinal permeability were lower in the RO group. Furthermore, the RO27-3225 treatment increased the expression of Rho-associated coiledecoil-containing protein kinase 1 and phosphorylated myosin light chain. Chl and HS024 abrogated the protective effects of RO27-3225.
[41]	Selective melanocortin 4 receptor agonist RO27-3225 (180 μg/kg ip) 2 min before blood was drawn. Selective melanocortin 4 receptor antagonist HS024 (130 μg/kg) 2 min before the RO27 3225 administration. The nicotinic acetylcholine receptor antagonist chlorisondamine (250 μg/kg) 2 min before the RO27 3225 administration	IAH rat models were induced by hemorrhagic shock/resuscitation (with the mean arterial pressure (MAP) maintained at 30 mm Hg for 90 min followed by the reinfusion of the withdrawn blood with lactated Ringer’s solution). Then, air was injected into the peritoneal cavity of the rats to maintain an intra-abdominal pressure of 20 mmHg for 4 h.	The permeability of the BBB, brain water content. The left brain hippocampus AQP4, MMP9, IL-1β and TNF-α concentrations were detected using an ELISA kit	The effects of the melanocortin 4 receptor agonist RO27-3225 in alleviating the rats’ IAH brain injuries were observed, which indicated that RO27-3225 could reduce brain edema, the expressions of the IL-1β and TNF-α inflammatory cytokines, the blood–brain barrier’s permeability and the aquaporin4 (AQP4) and matrix metalloproteinase 9 (MMP9) levels. Moreover, the nicotinic acetylcholine receptor antagonist chlorisondamine and the selective melanocortin 4 receptor antagonist HS024 can negate the protective effects of the RO27-3225.
[42]	Caffeic acid phenethyl ester (CAPE) at 10 μmol/kg was administered as a single intraperitoneal injection 1 h before the desufflation period	CO_2_ pneumoperitoneum 15 mmHg for 60 min.	The bronchoalveolar lavage was obtained twice with 3 mL of saline to investigate biochemical parameters, including paraoxonase (PON1) activity, total antioxidant status (TAS), total oxidative status (TOS) levels, and cytokine concentration.	CAPE to prevent CO_2_ pneumoperitoneum-induced oxidative stress and inflammatory reactions in lung tissue
[43]	Loading aprotinin dose of 28,000 KIU/kg ip straight after the onset of pneumoperitoneum, followed by lower maintenance doses (7500 KIU/kg), which were administered per hour until the termination of insufflation	Constant 12 mmHg pneumoperitoneum was maintained for 4 h. The duration of the reperfusion period was 60 or 180 min.	Several cytokines and markers of oxidative stress were measured in liver, small intestine, and lungs to compare the aprotinin group with the control group. Tissue inflammation was also evaluated and compared between groups using a five-scaled histopathologic score.	In the aprotinin group values of biochemical markers (tumor necrosis factor-a, interleukin 6, endothelin 1, C-reactive protein, pro-oxidant-antioxidant balance, and carbonyl proteins) were lower in all tissues studied. Statistical significance was greater in liver and lungs. Histopathologic examination revealed a significant difference between the control and aprotinin groups in all tissues examined. Aprotinin groups showed mild to moderate lesions, while in control groups, severe to very severe inflammation was present. The aprotinin subgroup with prolonged reperfusion period (180 min) showed milder lesions in all tissues than the rest of the groups.
[44]	Low-dose ketamine (KP1, 5 mg/kg; KP2, 10 mg/kg)	CO_2_ pneumoperitoneum of 15 mmHg.	Three hours after pneumoperitoneum, serum concentrations of interleukin-6 (IL-6), tumor necrosis factor-alpha (TNF-α), malondialdehyde (MDA), superoxide dismutase (SOD) and intestinal fatty acid binding protein (iFABP) were measured and liver, kidney, lung, and intestine were evaluated for tissue damage.	Pretreatment with low-dose ketamine before general anesthesia protects against potential oxidative damage and inflammatory response caused by CO_2_ pneumoperitoneum.
[45]	Intraperitoneal injection of caffeic acid phenethyl ester (CAPE) 10 μmol/kg one hour before the desufflation period	60 min of pneumoperitoneum with 15 mmHg IAP.	Kidneys, testicles, and prostate: Histology and the levels of the total oxidant status (TOS) and total antioxidant status (TAS)	In rat model, increased IAP had an oxidative effect on kidney and testis but not on prostate. Moreover, it could affect the testicular Johnsen score. According to kidney and testis tissues’ histologic evaluation, no significant alteration was obtained in 15 mmHg pressure groups for 1-h insufflation period. All these adverse effects of IAP on both kidney and testis could be prevented by CAPE administration. Further studies are needed to show oxidative effect of IAP against the tissues with more detailed morphological and biochemical analysis.
[46]	100 µg intraperitoneal dexmedetomidine 30 min before establishment of pneumoperitoneum.	60 min pneumoperitoneum was established under 12 mmHg pressure; intraperitoneal dexmedetomidine (100 µg) was administered 30 min before abdominal insufflation to establish 60 min pneumoperitoneum under 12 mmHg pressure.	Plasma total oxidant status (TOS), total antioxidant status (TAS), and oxidative stress index (OSI) activity were measured 30 min after the conclusion of pneumoperitoneum.	Dexmedetomidine decreases oxidative stress caused by pneumoperitoneum and strengthens the antioxidant defense system.
[47]	Dopamine infusion (3 μg/kg/min) before increasing IAP, a 60-min infusion of dopamine was performed; following this, IAP was raised, and the dopamine infusion (3 μg/kg/min) was continued for another 60 min.	IAP of 20 mmHg was maintained for 60 min by air insufflation	Renal artery perfusion was measured continuously for 30 min with a Doppler probe. Mean arterial pressure, myeloperoxidase (MPO) activity, lipid peroxidation and glutathione (GSH) levels were measured in tissue samples, and histopathological scoring was carried out.	Dopamine infusion before and during ACS, increases renal perfusion and decreases free oxygen radicals. Degenerations in the kidney tissues of the rats were clearly improved when the animals were treated by dopamine during and before ACS
[48]	Minocycline (20 mg/kg) was intravenously administered immediately after resuscitation.	Hemorrhagic shock/resuscitation was induced by blood drawing (mean arterial pressure: 40–45 mm Hg for 60 min) followed by shed blood/saline mixture reinfusion. Subsequently, intra-abdominal pressure (IAP) was increased to 25 mm Hg by injecting air into the preplaced intraperitoneal latex balloon to induce ACS. IAP was maintained at 25 mmHg for 6 h.	The levels of polymorphonuclear leukocyte infiltration, the wet/dry weight ratio, and the concentrations of inflammatory molecules (e.g., chemokine, cytokine, and prostaglandin E2) in lung and liver tissues	Minocycline ameliorates inflammatory response and organ dysfunction in the lungs and liver induced by hemorrhagic shock/resuscitation plus abdominal compartment syndrome, and ameliorated lung and liver injuries.
[49]	Dexmedetomidine administration (intraperitoneal injection of 100 mg/kg) 30 min before pneumoperitoneum.	Intra-abdominal pressure of 12 mmHg for 60 min. The rats were rested for 30 min after abdominal deflation.	Blood samples were obtained for plasma malondialdehyde and ischemia-modified albumin (IMA) analyses. Lung tissue samples were taken for histopathologic examination and malondialdehyde analysis.	Dexmedetomidine prophylaxis resulted in significantly less IMA production and significantly less neutrophil infiltration, thereby helping to protect the lungs from injury after pneumoperitoneum.
[50]	Tadalafil (10 mg/kg/day) for 4 days before the experiment	Rats with compensated and decompensated chronic heart failure (CHF) induced by the placement of an aorto-caval fistula (ACF), Rats with myocardial infarction induced by the left anterior descending (LAD) artery ligation and sham controls subjected to IAPs: 7, 10, 14 mmHg.	Urine flow rate (V), Na+ excretion (UNaV), glomerular filtration rate (GFR), renal plasma flow (RPF)	Amelioration of the adverse effects of high IAP
[51]	Two hours after operation, 10 mL/kg dachengqi tang (DCQT) was administered orally	Acute necrotic pancreatitis was induced by retrograde infusion of 5% taurocholic acid into the pancreatic duct.	Aterial blood, pancreas and lung tissues were collected for biomarkers and histopathology 24 h after operations. Intra-abdominal pressure and intestinal propulsion rate were also measured.	DCQT treatment reduced intra-abdominal pressure and improved intestinal propulsion rate compared with those treated with saline. The ANP rats treated with DCQT had a lower wet to dry weight ratio, and milder myeloperoxidase activity and histopathology changes in the pancreas and lung than those treated with saline. Higher pressure of oxygen (PO_2_) was found in the rats treated with DCQT.
[52]	Dopamine infusion (3 μg/kg/min) before increasing IAP, a 60-min infusion of dopamine was performed; following this, IAP was raised, and the dopamine infusion (3 μg/kg/min) was continued for another 60 min.	IAP of 20 mm Hg was maintained for 60 min by air insufflation.	Superior mesenteric artery (SMA) perfusion was measured continuously for 30 min with a Doppler probe. Mean arterial pressure, myeloperoxidase (MPO) activity, lipid peroxidation, and glutathione (GSH) levels were measured in tissue samples, and histopathological scoring was carried out.	Improved intestinal epithelium, improved glandular structure, SMA perfusion, counteracted hypotension, increased MPO activity, and decreased GSH.
[53]	Doxycycline (10 mg/kg i.p.) during the induction of ACS. Doxycycline (10 mg/kg i.p.) at 1 h after decompression	Intra-abdominal pressure at 20 mmHg by insufflating CO_2_ gas for 60 min. Decompression 1 h and 24 h.	Creatinine, kidney MDA, IL-1b, IL-6, TNF-a, MMP-2, and TIMP-1 were studied, and the apoptotic cells were enumerated histopathologically, and apoptosis and bcl-2 expression were immunohistochemically assessed.	Doxycycline had protective effects on I/R injury by decreasing apoptosis via reducing the level of pro-inflammatory cytokines, increasing the level of TIMP-1, and inhibiting the activity of MMP-2.
[54]	Glutamine through gastric gavage for 10 days at a dose of 1 mL per day (1 g/kg/day)	20 mmHg pressure was applied for 2 h using CO_2_.	Intestine, lung, and liver samples were removed for determination of tissue malondialdehyde (MDA) and glutathione (GSH) levels as oxidative injury parameters and of myeloperoxidase (MPO) activity as an inflammatory parameter. the alanine aminotransferase (ALT) and aspartate aminotransferase (AST) levels.	Glutamine decreased MDA levels and MPO activities and increased GSH levels.
[55]	Doxycycline (10 mg/kg IP) was injected during induction of ACS, and, similarly, intestinal samples were removed at 1 and 24 h after decompression.	IAP of 20 mmHg was maintained by insufflating with carbon dioxide gas for 60 min. Decompression 1 h and 24 h.	Intestine malondialdehyde (MDA), interleukin (IL)-1β, IL-6, tumor necrosis factor (TNF)-α, matrix metalloproteinase-2 (MMP-2), and tissue inhibitor of metalloproteinase-1 were studied and the apoptotic cells were enumerated histopathologically. Apoptosis and β-cell lymphoma 2 (βcl-2) expression were assessed immunohistochemically.	Doxycycline was associated with protective effects against I/R injury through decreasing apoptosis via attenuating the response of proinflammatory cytokines and inhibiting the activity of MMP-2 in this rat model.
[56]	Pentoxifylline (50 mg/kg ip) immediately before pneumoperitoneum.	CO_2_ pneumoperitoneum of 13 mmHg was established. At the first hour of insufflation, blood samples were taken to study the same parameters as in the control group. One hour following desufflation of CO_2_, blood samples were drawn again to study the same parameters.	The arterial pH, partial arterial oxygen pressure (PaO(_2_)), venous PO(_2_), arterial and venous PO(_2_) difference (P((a-v))O(_2_)), serum aspartate aminotransferase (AST), serum alanine aminotransferase (ALT), and thiobarbituric acid-reactive substances (TBARS) were studied at the end of the first and second hours	Pentoxifylline may reduce the oxidative injury following laparoscopic procedures.
[57]	Nitroglycerine (NTG) (i.v., prime 1.5 mg/kg and sustained infusion of 15 mg/kg/h) beginning 60 min before the application of 14 mmHg insufflation pressure for 1 h, followed by desufflation to 0 mmHg (recovery). L-arginine methylester (L-NAME) 100 mg/l added to drinking water for 4 days before the experiment.	IAP of 14 mmHg, over 1 h, followed by a deflation period of 1 h (recovery).	Urine flow rate (V), Na+ excretion (UNaV), glomerular filtration rate (GFR), renal plasma flow (RPF), and blood pressure	Counteraction of pneumoperitoneum-induced renal hypoperfusion and dysfunction (nitroglycerine). Aggravation of pneumoperitoneum-induced renal hypoperfusion and dysfunction (L-NAME)
[58]	Octreotide (50 µg/kg intraperitoneally) immediately before the decompression.	IAH 20 mmHg for 1 h, decompression 1 h.	ALT and AST levels, liver and intestinal tissues, malondialdehyde (an index of lipid peroxidation) and glutathione (a key to antioxidant) levels and myeloperoxidase (an index of tissue neutrophil infiltration) activity	Octreotide treatment reversed these oxidant responses, and reduced the elevations in both ALT and AST levels.
[59]	Melatonin (10 mg/kg, i.p.) immediately before the decompression of IAP.	IAH 20 mmHg for 1 h, decompression 1 h.	ALT and AST levels, liver and intestinal tissue, malondialdehyde (an index of lipid peroxidation) and glutathione (a key to antioxidant) levels and myeloperoxidase (an index of tissue neutrophil infiltration) activity, urea nitrogen (BUN), creatinine	Octreotide treatment reversed these oxidant responses, and reduced the elevations in ALT and AST, BUN and creatinine levels.
[60]	Octreotide (50 µg/kg intraperitoneally) immediately before the decompression	IAH 20 mmHg for 1 h, decompression 1 h.	Lung and kidney tissue, malondialdehyde (an index of lipid peroxidation) and glutathione (a key to antioxidant) levels and myeloperoxidase (an index of tissue neutrophil infiltration) activity urea nitrogen (BUN), creatinine	Octreotide treatment reversed these oxidant responses, and reduced the elevations In ALT and AST, BUN and creatinine levels.
[61]	Dopamine was dissolved in saline and applied as a continuous intravenous infusion at a rate of 3 µg/kg/min using a microperfusion pump with a volume of 2 mL/h. The selective endothelin-1 (ET-I) antagonist, JKC-30 l, was dissolved in saline and administered at 200 µg/kg intravenously as a single-shot injection.	Intraabdominal insufflation pressures were elevated in a stepwise manner from 2 to 12 mmHg (2, 4, 6, 8, 10, and 12 mmHg) every 10 min. At the end of the experimental procedure, the abdomen was desufltated and the portal venous blood flow recovery was measured for another 30 mm.	Portal blood flow was measured during intraabdominal pressures 2 to 12 mmHg.	Dopamine and ET-1 antagonism restore portal blood flow during laparoscopic surgery independently of the insufflation gas.
[62]	AVP V2 receptor antagonist, OPC-31260 (5 mg/kg) before pneumoperitoneum	1 h to carbon dioxide pneumoperitoneum (PP) with an intra-abdominal pressure of 8 mmHg.	Glomerular filtration rate (GFR). Urine output, excretion of water, and urea nitrogen, serum osmolality and serum sodium levels, blood urea nitrogen levels	OPC-31260 pretreatment did not affect GFR. Results suggest that plasma AVP contributes to the oliguria due to PP. OPC-31260 may be useful in treating the water retention associated with PP.

**Table 2:** Agents’ effectiveness in ACS and IAH in studies involving mice, rabbits, pigs, and dogs presented in the timeline. Studies included based on Pubmed search using the basic term “abdominal compartment syndrome, pigs”, “abdominal compartment syndrome, mice”, “abdominal compartment syndrome, rabbits”, and “abdominal compartment syndrome, dogs”.

Ref.	Agent/Species	IAH Procedure	Target	Outcome
[121]	Pigs. After 2 h of IAH, infusion of NO-donor PDNO (in a dose of 30 nmol kg^−1^ min^−1^) and the placebo drug was initiated and continued for 4 h until the experiment was ended.	IAH was induced by CO_2_ insufflation to 30 mmHg, after 2 h, decompression 4 h until the experiment was ended	Blood gases, invasive venous and arterial blood pressure, intestinal microcirculation and superior mesenteric blood flow were measured	NO-donor PDNO decreased systemic and pulmonary vascular resistances, elevated the cardiac index score, and, most importantly, counteracted decreased microcirculatory blood flow in the intestinal mucosa during IAH in a porcine model.
[122]	Mice. 120 μg (10 μL) hydromorphone 15 min before the establishment of pneumoperitoneum	Abdominal pressure to 15 mmHg for insufflation (for 1 h) and deflation with CO_2_ (for 3 h).	Lung injury myeloperoxidase (MPO), total oxidant status (TOS), and oxidative stress index (OSI), total antioxidant status (TAS). HO-1-regulated mitochondrial dynamics.	Hydromorphone alleviated lung injury in mice that underwent CO_2_ insufflation, decreased the levels of myeloperoxidase (MPO), total oxidant status (TOS), and oxidative stress index (OSI), and increased total antioxidant status (TAS). Hydromorphone protects against CO_2_ pneumoperitoneum-induced lung injury via HO-1-regulated mitochondrial dynamics.
[123]	Rabbits. Mydocalm (tolperison, 5 mg/kg single dose) at initiation of 3 h of IAH.	IAH level was modeled at 200 mmH_2_O with the subsequent stopping of further receipt of liquid during 3 h in an elastic container in the abdominal cavity.	Tone of muscles of the frontal abdominal wall, local blood flow, dilatation, constriction oxygen tension	Mydocalm reduces the tone of muscles of the frontal abdominal wall, which leads to a decrease in IAH (maximum effect after 1.5 h) and prevents decrease in the local blood flow, suppression of dilation and constriction, reactivity of vessels, and reduction in oxygen tension at the end of experiment.
[124]	Pigs. Ethyl nitrite (ENO) at a rate of 1.0 L/min in pigs using a high-flow clinical insufflator	The peritoneal cavity was next insufflated to a final intraperitoneal pressure of 15 mmHg with CO_2_ in the presence or absence of ENO at a rate of 1.0 L/min using a high-flow clinical insufflator. Pneumoperitoneum was maintained for 4 h during which gas was constantly vented from the peritoneal cavity to ensure continual CO_2_ and ENO exposure. After 4 h, insufflation was discontinued and the abdomen manually deflated. Monitoring was continued under anesthesia for an additional 2 h.	Regional tissue blood flow brain, heart, liver, kidney, stomach, small intestine, colon, spleen, pancreas, venous red blood cell SNO-Hb concentration	The data indicate ethyl nitrite can effectively attenuate insufflation-induced decreases in organ blood flow and nitric oxide bioactivity leading to reductions in markers of acute tissue injury.
[125]	Mice. Pentoxifyllin before IAH induction.	IAH was induced in mice by intraperitoneal infusion of mineral oil to a pressure of 20 mm Hg. 4 h of IAH followed by 1 h of decompression.	Brain blood barrier (BBB) integrity was determined by extravasation of 2% Evans blue (EB)	Pentoxifyllin improved BBB integrity
[126]	Mice. 50%, 80%, or 100% oxygen inhalation after establishing acute IAH	15, 20, 30, or 40 cmH_2_O IAP. The 40 cmH_2_O group seemed to be appropriate for follow-up experiments.	Liver and blood samples were used to compare the rates of apoptosis using the TUNEL assay as well as alanine aminotransferase (ALT), aspartate aminotransferase (AST); Caspase-3, 9, MDA, and SOD concentrations.	As the oxygen concentration increased, the survival time was prolonged among the 40 cmH_2_O IAP group, and the number of apoptotic hepatic cells decreased (P 0.01), with a concomittent decrease in caspase 3 and 9 as well as malondialdehyde, although superoxide dismutase showed the opposite results.
[127]	Dogs. Following preparation, fentanyl (1 μg kg(−1)) was injected over 30 s IV.	The abdomen was insufflated with CO(_2_) (11–16 cmH(_2_) O). Data were recorded 5 min before, during and 5 min after treatment. The following time points were selected for analysis: −160, −140, −120, −100, −80, −60, −40, −20, 0, 30, 50, 70, 90, 110, 130 and 150 s after the start of fentanyl injection.	Peak inspiratory and end-expiratory intra-abdominal pressures continuously decreased over time during the whole experiment and fentanyl exaggerated the decrease in inspiratory pressures but did not affect the rate of decrease in expiratory pressures.	Fentanyl did not increase intra-abdominal pressures in dogs.
[128]	Pigs. CO_2_ enriched with 100 ppm ethyl nitrite (ENO) in pigs.	Final intraperitoneal pressure of 15 mmHg. After 35 min, ENO was introduced into the peritoneal space by passing the CO_2_ gas insufflated for 60 min and then monitored for an additional 60 min after termination of insufflation	Liver and kidney blood flows.	Inclusion of ethyl nitrite (a nitric oxide-containing compound) within the insufflating gas significantly attenuated the decreases in liver blood flow produced during and after a 60-min period of carbon dioxide pneumoperitoneum.
[129]	Dogs. Enalaprilat (5 mg) 15 min before	Abdominal pressure was maintained automatically at 15 mmHg over a 60-min period. 15 and 30 min utes after deflation of the abdominal cavity	Body weight, hematologic values, hemodynamic parameters, and renal function (plasma renin activity, urinary debt, creatinine clearance, and sodium-excretory fraction)	The decline in urinary debt and in creatinine clearance observed during pneumoperitoneum was less accentuated with the administration of enalaprilat.
[130]	Pigs. CO_2_ was insufflated into the peritoneal cavity to reach an intraabdominal pressure of 15 mmHg. After 60 min, animals received dopamine (5 microg × kg(−1) × min(−1); n = 8), dobutamine (5 microg × kg(−1) × min(−1)).	CO_2_ was insufflated into the peritoneal cavity to reach an intraabdominal pressure of 15 mmHg.	Heart rate, mean arterial pressure, and systemic vascular resistance, with decreases in cardiac output and hepatic artery and portal vein blood flows	Dobutamine infusion, in contrast to dopamine, corrected, at least in part, cardiac output, systemic vascular resistance, and hepatic artery blood flow alterations, but neither drug restored total hepatic blood flow.
[131]	Pigs. CO_2_ was insufflated into the peritoneal cavity to reach an intra-abdominal pressure of 15 mmHg, and 60 min later, animals received dopamine (5 microg/kg/min; n = 10), dobutamine (5 microg/kg/min; n = 10), or saline (n = 5) for 30 min.	CO_2_ was insufflated into the peritoneal cavity to reach an intra-abdominal pressure of 15 mmHg, and 60 min.	A laser Doppler probe was positioned in the lumen of the ileum to measure arterial and intestinal mucosal blood flows.	Dobutamine infusion reversed the decrease in cardiac output, it failed to restore superior mesenteric artery blood flow; however, intestinal mucosal blood flow returned to baseline levels. Dopamine also attenuated the decrease in cardiac output, but it had no beneficial effect on splanchnic hemodynamic variables.
[132]	Pigs. The pneumoperitoneum was carefully evacuated, and after a l0-min rest, similar airway and abdominal pressure measurements were repeated after the pigs received a 4 mg/kg intravenous injection of atracurium.	Insufflation was discontinued when IAP was more than 15 mmHg.	Elastic properties of the abdomen (elastance).	High peak inspiratory airway pressures and intraabdominal pressures during laparoscopy are not affected by neuromuscular block.

## Data Availability

No new data were created or analyzed in this study. Data sharing is not applicable to this article.
